# Upregulation of the Chemokine Receptor CCR2B in Epstein‒Barr Virus-Positive Burkitt Lymphoma Cell Lines with the Latency III Program

**DOI:** 10.3390/v10050239

**Published:** 2018-05-03

**Authors:** Svetlana Kozireva, Zhanna Rudevica, Mikhail Baryshev, Ainars Leonciks, Elena Kashuba, Irina Kholodnyuk

**Affiliations:** 1 August Kirchenstein Institute of Microbiology and Virology, Riga Stradins University, 5 Ratsupites Str, 1067 Riga, Latvia; svetlana.kozireva@rsu.lv (S.K.); mihails.barisevs@rsu.lv (M.B.); 2 Latvian Biomedical Research and Study Centre, 1 Ratsupites Str k-1, 1067 Riga, Latvia; zh_rudevica@inbox.lv (Z.R.); ainleo@biomed.lu.lv (A.L.); 3 Department of Microbiology, Tumor and Cell Biology (MTC), Karolinska Institutet, 16 Nobelsväg, Box 280, 171 77 Stockholm, Sweden; elena.kashuba@ki.se; 4 R.E. Kavetsky Institute of Experimental Pathology, Oncology, and Radiobiology, NASU, 45 Vasylkivska str, 03022 Kyiv, Ukraine; Kashuba@nas.gov.ua

**Keywords:** CCR2, EBV, latency III, Burkitt lymphoma cell lines, B-cell lymphoma

## Abstract

CCR2 is the cognate receptor to the chemokine CCL2. CCR2–CCL2 signaling mediates cancer progression and metastasis dissemination. However, the role of CCR2–CCL2 signaling in pathogenesis of B-cell malignancies is not clear. Previously, we showed that CCR2B was upregulated in ex vivo peripheral blood B cells upon Epstein‒Barr virus (EBV) infection and in established lymphoblastoid cell lines with the EBV latency III program. EBV latency III is associated with B-cell lymphomas in immunosuppressed patients. The majority of EBV-positive Burkitt lymphoma (BL) tumors are characterized by latency I, but the BL cell lines drift towards latency III during in vitro culture. In this study, the CCR2A and CCR2B expression was assessed in the isogenic EBV-positive BL cell lines with latency I and III using RT-PCR, immunoblotting, and immunostaining analyses. We found that CCR2B is upregulated in the EBV-positive BL cells with latency III. Consequently, we detected the migration of latency III cells toward CCL2. Notably, the G190A mutation, corresponding to SNP CCR2-V64I, was found in one latency III cell line with a reduced migratory response to CCL2. The upregulation of CCR2B may contribute to the enhanced migration of malignant B cells into CCL2-rich compartments.

## 1. Introduction

Epstein‒Barr virus (EBV) is a human gammaherpesvirus 4, which establishes a life-long latent infection in memory B cells and can be reactivated under immunosuppression [1]. The virus is associated with various cancers, including B-cell malignancies such as Burkitt lymphoma (BL), a set of diffuse large B cell lymphomas (DLBCL), and post-transplant and immunodeficiency-related lymphomas (reviewed in [2,3,4,5,6]). EBV encodes several latent proteins and can affect the expression of different cellular genes depending on its latency program. Four EBV infection latency types are known (I, IIA, IIB, and III) [7,8,9,10]. EBV-encoded nuclear protein 1 (EBNA1), which is expressed in all EBV latency programs, is responsible for episome replication and the maintenance of latent infection. EBNA1 can upregulate the STAT1 (signal transducer and activator of transcription 1) protein and downregulate SMAD2 (SMAD family member 2) and tyrosine phosphatase receptor K, and inhibit phosphorylation of p65 kinase (reviewed in [10]). EBNA2 and EBNA-LP are the first proteins to be coexpressed after the infection of B cells. EBNA2 can activate the orphan nuclear receptor NUR77, which enhances expression of the BCL family protein BFL/A2, reduces expression of the death-inducer BIK, and participates in the upregulation of *MYC* (reviewed in [10,11]). EBNA3C was demonstrated to be involved in the stabilization of IRF4 and upregulation of Pim1 kinase. EBNA1, EBNA2, EBNA3A, EBNA3B, EBNA3C, and EBNA-LP are expressed in the latency III program. EBNA3A and EBNA3C can downregulate the expression of tumor suppressors p14ARF and p16INK4A, and the chemokine receptor CXCR10, while EBNA3B can inhibit cell growth and upregulate CXCR10 (reviewed in [8,10]). EBNAs expression is followed by expression of the latent membrane proteins (LMPs). LMP1, a major viral oncogene, is essential for transformation of B cells. Induction of various cellular factors, including CD40, ICAM1, CD21, and LFAI, by LMP1 and its implication in activation of the NF-ĸB-, ERK-, JNK-, and p38-signaling pathways via the upregulation of prosurvival proteins, such as BCL-2 and MCL1, and the chemokines, CCL3 and CCL4, was reported previously (reviewed in [10,11,12,13]). Latency I, in which only the EBNA1 protein is expressed, is a typical feature of EBV-positive BL tumors (reviewed in [1,2,3,4,5,6]). However, following the cultivation in vitro, BL cell lines can drift towards the latency III program (reviewed in [1,2,3,4]).

EBV latency III infection activates B cells, which induce cell surface antigens and adhesion molecules [14,15,16,17]. Increased expression of CCR6 and CCR10 was detected in human EBV-immortalized B cells, but not in the EBV-positive BL cell lines with latency I. The authors also demonstrated that expression of EBNA2 in the EBNA2-transfected EBV-negative B-cell line BJAB induced CCR6 but not CCR10 expression [18]. The upregulation of *CCR2* and *CCR9* mRNA expression levels was also shown in tonsillar B cells after EBV infection in vitro [19].

Chemokines and their receptors are the major players in both innate and adaptive immunity; they promote migration of immune cells toward a site of infection and inflammation (reviewed in [20,21]. Chemokine receptors are G protein-coupled proteins composed of seven helical transmembrane loops. Approximately 20 chemokine receptors are known in mammalians. Most of the chemokine receptors are selective for chemokines of one subfamily, and are named and classified according to the subfamily of ligand chemokines [22].

CCL2, which is also known as monocyte chemoattractant protein 1 (MCP1), is the cognate (dominant) ligand for CCR2, although CCL2 can bind to CCR3 and CCR5 in the absence of the cognate receptor CCR2 [22,23]. CCR2, CCR1, CCR3, and CCR5 belong to the same protein sequence homology cluster, i.e., they have high protein sequence identity and can bind the same chemokines. Most chemokine receptors can respond to multiple nondominant chemokines in the absence or inaccessibility of the cognate ligand (reviewed in [21,22]). Notably, the *CCR1, CCR2, CCR3*, and *CCR5* genes reside in the same region at human 3p21.31 [24]. CCR2 can bind other chemokines, such as CCL7, CCL8, and CCL13. Binding of different chemokines to the same receptor can result in distinct biological reactions (reviewed in [20,22]). Numerous studies demonstrated that CCR2–CCL2 signaling mediates and stimulates cancer progression and metastasis dissemination (reviewed in [21,25,26]. However, the role of CCR2–CCL2 signaling in B-cell malignancies is largely unknown.

CCR2 exists in two isoforms, CCR2B and CCR2A, which differ in their C-terminal region [21,22]. Recently, we reported that costimulation with the CD40 ligand (anti-CD40 antibodies) and interleukin 4, as well as EBV infection, upregulated the expression of CCR2B, but not CCR2A, in peripheral blood (PB) B cells isolated from healthy donors. The enhanced *CCR2B* mRNA expression level was maintained in the established lymphoblastoid cell lines (LCLs) with the EBV latency III program [27]. The present study was focused on CCR2, the dominant receptor for CCL2 (MCP1), and its status in the isogenic EBV-negative and EBV-positive BL cell lines expressing EBV latency I and III programs to verify the impact of EBV infection on CCR2 upregulation.

## 2. Materials and Methods

### 2.1. Cell Lines

Two sets of isogenic BL cell lines from the cell line collection at MTC, Karolinska Institute (Stockholm, Sweden) were studied. The Mutu cell lines were generated from an EBV-carrying early passage BL cell line by in vitro culture and clone selection. Mutu cl.148 with EBV latency I had a group I phenotype, while Mutu cl.99 with EBV latency III had a group III phenotype [16]. Mutu III was derived from the latency I Mutu clone cell line that acquired EBV latency III in vitro [28]. Mutu cl.30, the EBV-negative BL line, was also derived from the parental latency I Mutu cells [29]. The EBV-positive Mutu cell lines carry EBV strain type 1. The BL cell lines, Jijoye M13 (latency I) and Jijoye P79 (latency III), carry EBV strain type 2 [29,30]. Cells were cultured in Iscove’s modification of Dulbecco’s medium (IMDM) with 10% fetal bovine serum (FBS) and harvested for analyses on the following day after cell splitting.

### 2.2. Detection of the CCR2 and EBV Gene Expression Using Real-Time RT-PCR

Total RNA was isolated from the cell lines using TRI Reagent (Sigma-Aldrich, Steinhem, Germany). A cDNA was transcribed with the random (N)_6_ primer using a cDNA synthesis kit (Thermo Fisher Scientific, Waltham, MA, USA). The resulting cDNA (corresponding to 100 ng of total RNA equivalent) was amplified in triplicate, using PerfeCTa SYBR Green FastMix (Quanta BioSciences Inc., Beverly, MA, USA), with the *CCR2*-specific primers overlapping the first and second exons and also with the specific primers for the house-keeping gene *TBP* (encoding TATA box-binding protein), as described previously [27]. The expression of two EBV-encoded latent genes (*EBNA2* and *LMP1*) was examined as described above for *CCR2*. The real-time PCR analyses were performed using the CFX96 Touch Real-Time PCR Detection System (Bio-Rad Laboratories Inc., Richmond, CA, USA). The primer sequences for *EBNA2* type 1 and *LMP1* were described previously [31]. The *EBNA2* transcript in the Jijoye M13 and Jijoye P79 cell lines was determined using the EBV type 2-specific primers [32]. Melting curve analysis was performed to confirm the specific products.

### 2.3. Detection of the CCR3, CCR5, CCR2A, and CCR2B Open Reading Frame (ORF) Transcripts Using RT-Duplex-PCR and Sequencing

The *CCR2A* and *CCR2B* ORF-transcripts were determined using RT-duplex-PCR with two pairs of primers, flanking the *CCR2A* or *CCR2B* ORF and corresponding to the housekeeping gene *GAPDH*. We described the RT-duplex-PCR protocol and primer sequences previously [27]. We also published the primers used for the amplification of the *CCR3* (exon 1–4 in both variants 1 and 2) and *CCR5* (exon 2–3) transcripts previously [33]. For RT-duplex-PCR, purified total RNA was reverse transcribed using RevertAid First Strand cDNA Synthesis Kit with the oligo(dT)_18_ primer (Thermo Fisher Scientific).

The *CCR2B* ORF PCR-products from BL cell lines were gel-purified and cloned using the TOPO TA cloning kit (Thermo Fisher Scientific). To prevent clonal amplification of sequences, the transformed competent cells were plated immediately after the heat shock, excluding shaking bacteria for 1 hour. Five clones for each PCR product were sequenced using the BigDye Terminator sequencing kit (v. 3.1; Thermo Fisher Scientific). The mutation analysis of *CCR2B* ORF was performed by alignment of the obtained sequences against the *CCR2B* mRNA RefSeq (NM_001123396.2). Nucleotide substitutions were accepted when they were present in the same position in all five clones.

### 2.4. Detection of CCR2 Using Western Blot Analysis

Total cell lysates in Laemmli buffer were prepared: each well was loaded with the lysate made of 2 × 10^5^ cells. Proteins were separated using SDS polyacrylamide gel electrophoresis and transferred onto a nitrocellulose membrane. The CCR2 protein was detected using immunoblotting with the rabbit monoclonal anti-CCR2 antibody (clone E68; Novus Biologicals, Littleton, CO, USA). The LCL that was established by infection of ex vivo PB B cells with the EBV strain B95-8, and the human myeloid histiocytic lymphoma cell line U937 was used as the positive control. For detection of the EBV antigens EBNA2 and LMP1, mouse monoclonal antibody PE-2 (anti-EBNA2; DAKO, Copenhagen, Denmark) and NCL-EBV-CS1-4 (anti-LMP1; Novocastra Laboratories, Newcastle upon Tyne, UK) were used.

### 2.5. Detection of CCR2B Using Fluorescent Immunostaining Analysis

The cells were fixed in the methanol–acetone mixture (1:1) at −20 °C. Immunostaining with the mouse monoclonal anti-CCR2B antibody (CKR-2B, sc-74490; Santa Cruz Biotechnology, Inc., Dallas, TX, USA) and the FITC-conjugated rabbit anti-mouse antibody was performed as described previously [17]. Hoechst 33258 (Sigma-Aldrich) was added at a concentration of 0.4 μg/mL in the secondary antibody solution for DNA nuclear counterstaining. The Eclipse 80i Nikon microscope (Nikon Instruments Europe, Amsterdam, The Netherlands) was used to capture the images.

### 2.6. Cell Migration Assay

A cell migration assay was performed using the HTS Transwell-96 Well Permeable Support System (Corning Incorporated Life Sciences, Cambridge, MA, USA), with pore size 8 or 5 μm. Before the chemotactic migration experiment, cells were washed and cultured in a serum-free medium at 37 °C in 5% CO_2_ for 3 h. 10^5^ cells in 100 μL of the chemotaxis medium (CM, phenol-red, and serum free IMDM medium supplemented with 0.5% BSA) were loaded into each upper well to account for the chemotactic migration of cells toward different concentrations of human recombinant MCP1/CCL2 (BioLegend, San Diego, CA, USA): 50, 100, 200, and 1000 ng/mL of CM (100 μL). CM and 7% FBS in CM were used as negative and positive controls. After incubation at 37 °C in 5% CO_2_ for 4 h, the cells were collected in the lower wells were assessed using the MTT test (Vybrant MTT Cell Proliferation Assay Kit; Thermo Fisher Scientific). GraphPad Prism software (v.7; GraphPad Software, La Jolla, CA, USA) was used for multiple comparisons of nonparametric criteria for the experimental data. Two-way ANOVA was applied to analyze the mean differences between the relative migration of the cells and two independent variables, i.e., the CCL2 concentration and cell type.

## 3. Results

**Figure 1:**
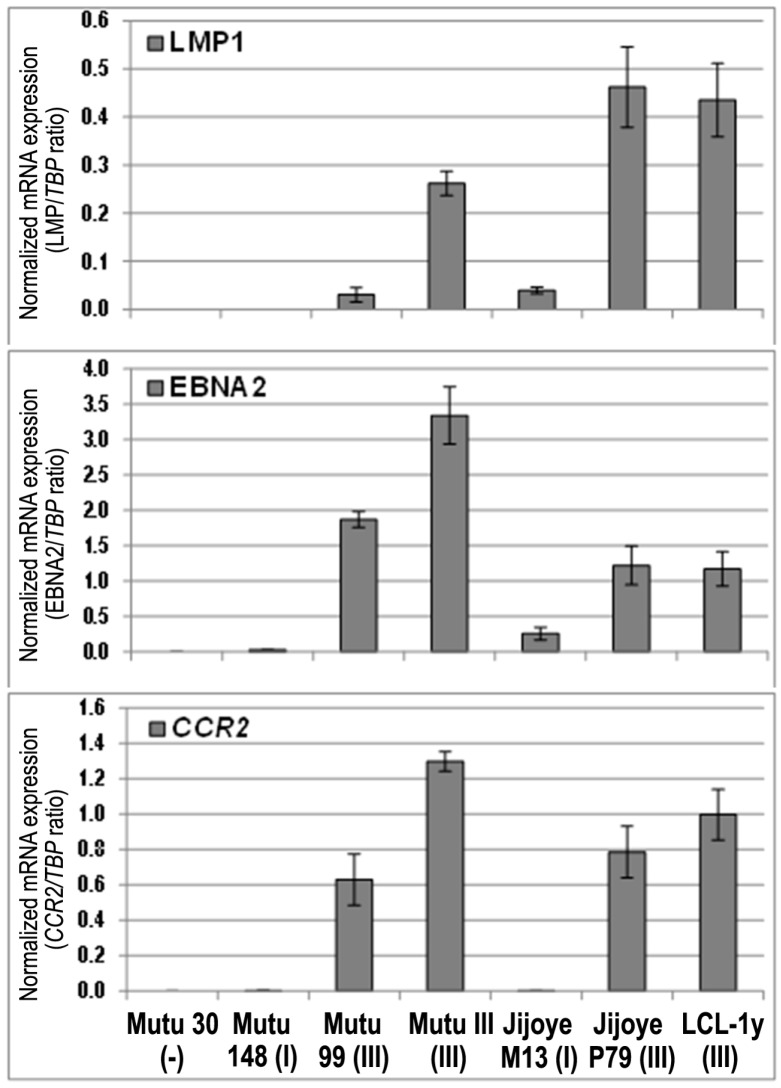
The *CCR2* and Epstein‒Barr virus (EBV) genes, EBNA2 and LMP1, mRNA expression in the isogenic BL cell lines, Mutu and Jijoye, with the EBV latency I and III programs. The *CCR2*, EBNA2, and LMP1 mRNA expression levels were normalized to the house-keeping gene *TBP* mRNA expression level for each sample and the ratio values are shown. LCL-1y is a LCL cell line included as a positive control. Mutu 30 (-) is the EBV-negative Mutu 30 cell line. Error bars show SD.

The *CCR2* mRNA expression was detected using real-time RT-PCR with the specific primers for both isoforms in the EBV-positive BL cell lines with latency III, while the latency I BL cell lines showed very low expression levels (Figure 1). The *CCR2* mRNA expression (the *CCR2*/*TBP* mRNA expression ratio) in the Mutu cell lines with latency III, Mutu III and Mutu cl.99, was 806- and 392-fold higher, respectively, compared to the latency I Mutu cl.148 cell line. The *CCR2*/*TBP* expression ratio in the Jijoye cells with latency III, Jijoye P79, was 1495-fold higher in comparison with the latency I Jijoye M13 cells. At the same time, the EBNA2/*TBP* mRNA ratio in Mutu III and Mutu cl.99 cells were 132- and 74-fold higher than the ratio in Mutu cl.148 with latency I, respectively; in Jijoye P79, a 4.8-fold increase only compared to that in latency I Jijoye M13. The LMP1/*TBP* mRNA ratio was 8.7-fold higher in Mutu III cells compared to Mutu cl.99 cells. The LMP1 transcript was not found in Mutu cl.148 cells (latency I). The LMP1 mRNA expression was determined in both Jijoye cell lines, although the LMP1/*TBP* mRNA ratio was 11.7-fold higher in the latency III Jijoye P79 cells. We should note that the BL cell line Jijoye M13 was previously characterized as an EBV-positive BL cell line with the latency I program [30]. In the present study, the EBNA2 and LMP1 transcripts were detected at low level in this cell line, indicating a shift towards latency III. Nonetheless, *CCR2* mRNA expression was correlated with EBNA2 expression (Figure 1).

**Figure 2:**
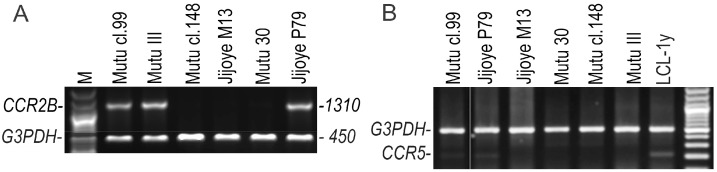
The *CCR2B* ORF (**A**) and *CCR5* (**B**) transcripts in the BL cell lines with latency III were detected by RT-duplex-PCR. BL cell lines with latency III: Mutu III, Mutu cl.99, and Jijoye P79; BL cell lines with latency I: Mutu cl.148 and Jijoye M13; Mutu 30, the EBV-negative BL cell line. M, the GeneRuler 100 bp Plus DNA Ladder (Thermo Fisher Scientific).

The *CCR2B* ORF-transcript was determined in all BL cell lines with latency III using RT-duplex-PCR (Figure 2A). The *CCR2A* ORF-transcript was not detected in any of the cell lines in this study. At very low levels, the *CCR3* and *CCR5* transcripts were found by RT-duplex-PCR in only two latency III BL cell lines, Mutu cl.99 and Jijoye P79, but not in Mutu III (Figure 2B). Thus, this excludes their possible involvement in the chemotactic response to CCL2.

The analysis of sequences of the *CCR2B* ORF-transcripts revealed a single-nucleotide nonsynonymous mutation (G190A) resulting in substitution of valine to isoleucine (V64I) in one of the Mutu cell lines, Mutu cl.99. This mutation corresponds to the known single-nucleotide polymorphism (SNP) CCR2-V64I.

**Figure 3:**
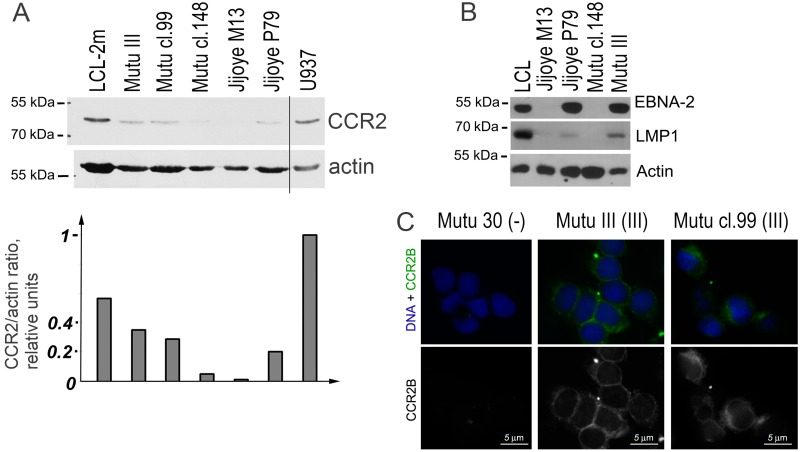
CCR2 protein expression in the EBV-positive Burkitt lymphoma (BL) cell lines. Immunoblotting analyses of BL cell lines Mutu III (latency III), Mutu cl.99 (latency III), Mutu cl.148 (latency I), Jijoye M13 (latency I), Jijoye P79 (latency III) for detection of the CCR2 protein (**A**) and the EBV antigens EBNA2 and LMP1 (**B**). The LCL cell line with latency III (LCL-2m) and the human myeloid histiocytic lymphoma cell line U937 are used as the positive controls. (**C**) CCR2B was determined by immunofluorescence microscopy using immunostaining with the mouse monoclonal anti-CCR2B antibody. The nuclear DNA staining with Hoechst is shown in blue. Mutu 30 (-) is the EBV-negative BL line Mutu 30. Scale bar = 5 μm.

The immunoblotting analysis showed that all BL cell lines with latency III expressed the CCR2 protein (Figure 3A). A very weak signal can be also seen in the cells with latency I. Immunostaining demonstrated the membrane localization of the CCR2B protein in the latency III cells (Figure 3B).

**Figure 4:**
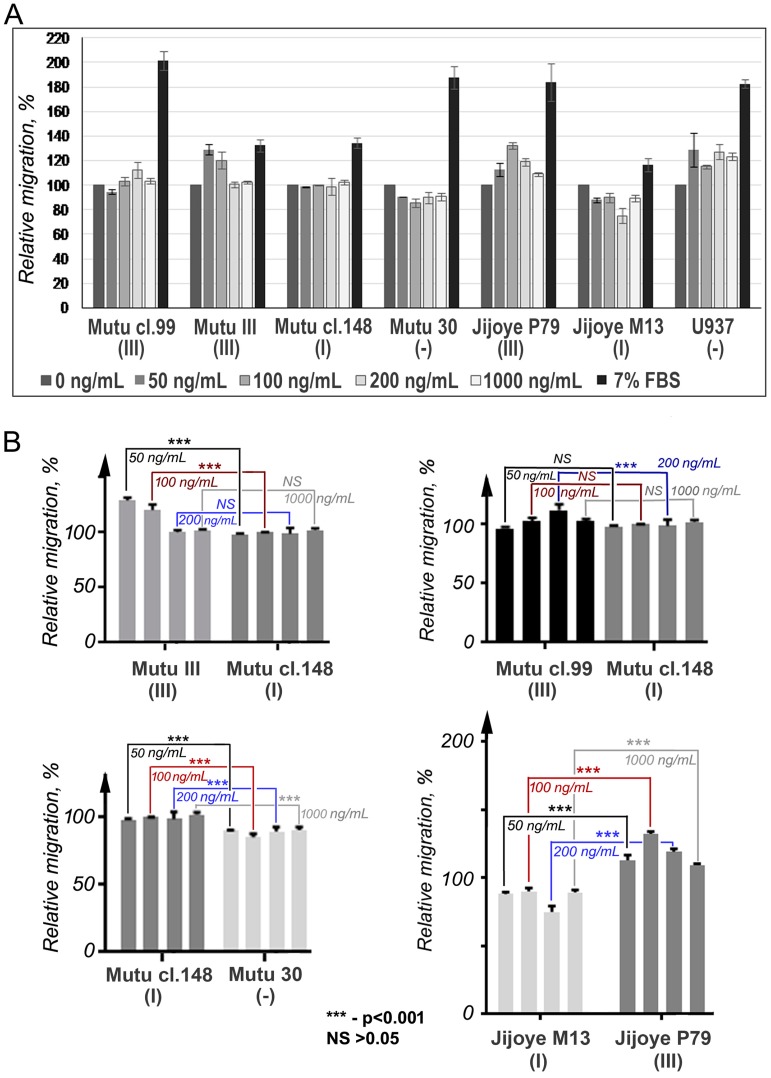
Chemotactic migration of BL cells with latency III in response to CCL2. Mutu cl.99, Mutu III, and Jijoye P79 are BL cell lines with latency III; Mutu cl.148 and Jijoye M13 with latency I; and Mutu 30, the EBV-negative BL cell line. U937 (ATCC CRL 1593), the EBV-negative human histiocytic lymphoma cell line, was included as a positive control. For U937 cells, the assay was conducted using a membrane with 5-μm pores. (**A**) The results are shown as the relative migration (%): the values of migration into the MCP1-free chemotaxis medium were set as 100. The data are presented as the mean and the SD errors of duplicate samples in two independent experiments. (**B**) Significant differences in the migratory activities were defined in the pair-wise comparisons: Mutu cl.148 cells versus Mutu III (for concentrations 50 and 100 ng/mL), *p* < 0.001 (**the top left panel**); Mutu cl.148 cells versus Mutu cl.30 (for all four concentrations), *p* < 0.001 (**the bottom left panel**); Jijoye M13 versus Jijoye P79 (for all four concentrations), *p* < 0.001 (**the right bottom panel**). No significant differences were detected when comparing Mutu cl.148 and Mutu cl.99 at all concentrations, except one, the 200 ng/mL (**the right top panel**).

To find out whether CCR2 is functional in BL cell lines with latency III, the chemotactic migration (chemotaxis) assay, using the human recombinant chemokine CCL2, was performed (Figure 4). Migration of the cells with latency I and EBV-negative in response to CCL2 was not determined. Cells from the latency III BL cell lines migrated toward the CCL2, although the Mutu cl.99 cells showed significantly lower, if at all, migration that can be putatively connected with the mutation G190A found within the *CCR2B* ORF transcript. The reduced migration of the CCR2-positive cells was observed in the presence of CCL2 at high concentrations, which was likely due to the fast saturation and consequent internalization of the receptor [21,22].

## 4. Discussion

Our results have shown that only the EBV-positive BL cells with latency III expressed the functional CCR2B protein. Moreover, the *CCR2* mRNA level correlated with the EBNA2 mRNA expression. We may speculate that EBNA2 can be directly involved in the CCR2B upregulation in cells with EBV latency III infection. Recently, we have reported that CCR2B, but not CCR2A, was induced in PB B cells upon EBV infection in vitro and maintained its upregulation in the established LCLs with EBV latency III [27]. The EBV-infected latency III B cells can be found in vivo. However, in immunocompetent persons, such cells are eliminated by cytotoxic T cells because of the high immunogenicity of the infected cells [1]. CCR2 is not only the cognate (dominant) receptor for CCL2/MCP1, it binds and responds to several inflammatory chemokines, such as CCL7/MCP3, CCL8/MCP2, CCL13/MCP4, and CCL16/MTN-1, which are secreted by inflammatory macrophages, monocytes, and cytotoxic T cells [34]. We suggested earlier that the expression of CCR2B on EBV-infected B cells with latency III in immunocompetent persons might be involved in recognition and elimination of those cells by cytotoxic immune cells [27]. Indeed, B-cell lymphoproliferative disorders (LPDs), which are associated with the EBV latency III program, are preferentially found in patients with immunodeficiency, e.g., HIV-related and post-transplant lymphoproliferative disease (reviewed in [1,5,6]).

Here, we also report the single-nucleotide mutation corresponding to the known CCR2-V64I SNP that we detected in one BL cell line with latency III (Mutu cl.99). These cells demonstrated significantly lower chemotactic migration toward the CCL2. The correlation between the mutation G190A (CCR2-V61I) and the response to CCL2 remains to be verified. The CCR2-V64I variant in the transmembrane region can limit responses of other receptors, e.g., the CCR2-V64I variant had no influence on the CCR2B expression in T cells while the CXCR4 expression in these cells was slightly lower [35]. The heterodimerization between CCR2 and CCR5 was described previously [35]. Transinhibition of ligand binding for CCR2/CXCR4 was demonstrated in transfected and primary leukocytes. For the CCR2/CCR5/CXCR4 multimer, this effect was shown in transfected cells, primary T cells, and monocytes [36]. The synergistic effect, after the simultaneous activation of two receptors, was observed in embryonic kidney cells that were transfected with plasmids encoding *CCR2B* and *CCR5*. Upon the simultaneous stimulation of the transfected cells with CCL2/MCP1 and CCL5/RANTES, Ca^2+^ flux was induced by much lower concentrations of these chemokines than were necessary for induction by one chemokine [37]. Therefore, the CCR2-V64I variant might affect responses of other receptors expressed on B cells. Notably, the CCR2-V64I polymorphism, found in DNA isolated from the PB of patients with various cancers, was associated with the increased risk of cancer development [38,39,40].

The CCR2–CCL2 axis has been implicated in the pathogenesis of several diseases, including cancer, allergy, inflammatory, and autoimmune diseases. Enhanced levels of CCL2 in a variety of tumors were associated with cancer progression and increased tumor growth. Not only tumor cells, but also the surrounding non-neoplastic stroma express high levels of CCL2, which mediates the recruitment of T lymphocytes, fully differentiated M2 macrophages, and circulating monocytic cell subsets into the tumor environment (reviewed in [21,25,26]). Macrophages and circulating monocytes constitutively express CCR2 while its expression in T cells is inducible (reviewed in [21,22]). In transient immunosuppression, the EBV-infected CCR2B-expressing latency III B cells may be attracted into the tumor-surrounding stroma, where the virus can spread and infect the neighboring cells.

EBV is transmitted in humans by saliva. The secretion of the virus into saliva is well documented [41,42] (reviewed in [1]), which suggests an involvement of the oral epithelium. Indeed, cell-free EBV enters polarized oral epithelial cells, which can lead to a lytic productive infection [43,44]. Furthermore, EBV can transmigrate across oral epithelial cells by transcytosis; i.e., the transport of virions from one membrane to another through the cytoplasm without infecting cells [45]. Notably, the expression of CCL2 (MCP1) and CCL5 (RANTES) in EBV-negative epithelial keratinocyte cells in vitro can be induced by the CD40 ligation or by ectopic expression of LMP1 [46]. The ligand of the constitutively expressed CD40 is presented by T cells. The transmission of EBV to monocytes was demonstrated in vitro by the cocultivation of EBV-infected B cells with monocytes [47], which suggests that EBV-infected B lymphocytes can be the source of a viral infection of intraepithelial macrophages and dendritic cells. The EBV infection of macrophages, monocytes, and dendritic cells has been shown in vitro and in vivo [48,49]. Thus, a possible mechanism of EBV spread is that EBV-infected CCR2-expressing B lymphocytes migrate into the CCL2-expressing oral epithelium where they reproduce the cell-free virus via lytic replication. The virus infects the intraepithelial macrophages and dendritic cells, and, stimulated by factors released by the intraepithelial immune cells, transmigrates through the uninfected oral epithelial cells into the saliva.

We showed that EBV upregulates CCR2B expression in BL cells with latency III. The upregulation of CCR2B in these malignant B cells enhances their migration toward CCL2. Since immunodeficiency-related B-cell LPDs, including the post-transplant lymphoma and subsets of DLBCL, are characterized by EBV latency III, we suggest that the EBV-encoded latency III proteins may be involved in the regulation of CCL2–CCR2 signaling, which is a part of the immune-response machinery, thus affecting the pathogenesis of these malignancies.

## References

[1] Young L.S., Yap L.F., Murray P.G. (2016). Epstein-Barr virus: More than 50 years old and still providing surprises. Nat. Rev. Cancer.

[2] Bornkamm G.W. (2009). Epstein-Barr virus and the pathogenesis of Burkitt’s lymphoma: More questions than answers. Int. J. Cancer.

[3] Klein G., Klein E., Kashuba E. (2010). Interaction of Epstein-Barr virus (EBV) with human B-lymphocytes. Biochem. Biophys. Res. Commun..

[4] Klein G. (2009). Burkitt lymphoma—A stalking horse for cancer research?. Semin. Cancer Biol..

[5] Ok C.Y., Li L., Young K.H. (2015). EBV-driven B-cell lymphoproliferative disorders: From biology, classification and differential diagnosis to clinical management. Exp. Mol. Med..

[6] Shanon-Lowe C., Rickinson A.B., Bell A.I. (2017). Epstein-Barr virus-associated lymphomas. Philos. Trans. R. Soc. Lond. B..

[7] Klein E., Nagy N., Rasul A.E. (2013). EBV genome carrying B lymphocytes that express the nuclear protein EBNA-2 but not LMP-1: Type IIb latency. Oncoimmunology.

[8] Niller H.H., Szenthe K., Minarovits J. (2014). Epstein-Barr virus-host cell interactions: An epigenetic dialog?. Front. Genet..

[9] Price A.M., Luftig M.A. (2015). To be or not IIb: A multi-step process for Epstein-Barr virus latency establishment and consequences for B cell tumorigenesis. PLoS Pathol..

[10] Kang M.S., Kieff E. (2015). Epstein-Barr virus latent genes. Exp. Mol. Med..

[11] Kempkes B., Robertson E.S. (2015). Epstein-Barr virus latency: Current and future. Curr. Opin. Virol..

[12] Hatton O.L., Arnold-Harris A., Schaffert S., Krams S.M., Martinez O.M. (2014). The Interplay between Epstein Barr Virus and B lymphocytes: Implications for infection, immunity, and disease. Immunol. Res..

[13] Wang L.W., Jiang S., Gewurz B.E. (2017). Epstein-Barr virus LMP1-Mediated oncogenicity. J. Virol..

[14] Rowe M., Rowe D.T., Gregory C.D., Young L.S., Farrell P.J., Rupani H., Rickinson A.B. (1987). Differences in B cell growth phenotype reflect novel patterns of Epstein-Barr virus latent gene expression in Burkitt’s lymphoma cells. EMBO J..

[15] Gregory C.D., Kirchgens C., Edwards C.F., Young L.S., Rowe M., Forster A., Rabbitts T.H., Rickinson A.B. (1987). Epstein-Barr virus-transformed human precursor B cell lines: Altered growth phenotype of lines with germ-line or rearranged but nonexpressed heavy chain genes. Eur. J. Immunol..

[16] Gregory C.D., Rowe M., Rickinson A.B. (1990). Different Epstein-Barr virus-B cell interactions in phenotypically distinct clones of a Burkitt’s lymphoma cell line. J. Gen. Virol..

[17] Rincon J., Prieto J., Patarroyo M. (1992). Expression of integrins and other adhesion molecules in Epstein-Barr virus-transformed B lymphoblastoid cells and Burkitt’s lymphoma cells. Int. J. Cancer.

[18] Nakayama T., Fujisawa R., Izawa D., Hieshima K., Takada K., Yoshie O. (2002). Human B cells immortalized with Epstein-Barr virus upregulate CCR6 and CCR10 and downregulate CXCR4 and CXCR5. J. Virol..

[19] Ehlin-Henriksson B., Liang W., Cagigi A., Mowafi F., Klein G., Nilsson A. (2009). Changes in chemokines and chemokine receptor expression on tonsillar B cells upon Epstein-Barr virus infection. Immunology.

[20] White G.E., Iqbal A.J., Greaves D.R. (2013). CC Chemokine receptors and chronic Inflammation—Therapeutic Opportunities and Pharmacological Challenges. Pharmacol. Rev..

[21] Zabel B.A., Rott A., Butcher E.C. (2015). Leukocyte chemoattractant receptors in human disease pathogenesis. Annu. Path. Mech. Dis..

[22] Stone M.J., Hayward J.A., Huang C., Huma Z.E., Sanchez J. (2017). Mechanisms of Regulation of the Chemokine-Receptor Network. Int. J. Mol. Sci..

[23] The IUPHAR/BPS Guide to PHARMACOLOGY Database. http://www.guidetopharmacology.org/.

[24] Kholodnyuk I., Szeles A., Yang Y., Klein G., Imreh S. (2000). Inactivation of the human *Fragile Histidine Triad* gene at 3p14.2 in monochromosomal human/mouse microcell hybrid-derived severe combined immunodeficient mouse tumors. Cancer Res..

[25] Lim S.Y., Yuzhalin A.E., Gordon-Weeks A.N., Muschel R.J. (2016). Targeting the CCL2-CCR2 signaling axis in cancer metastasis. Oncotarget.

[26] Yoshimura T. (2017). The production of monocyte chemoattractant protein-1 (MCP-1)/CCL2 in tumor microenvironments. Cytokine.

[27] Kholodnyuk I., Rudevica Z., Leonciks A., Ehlin-Henriksson B., Kashuba E. (2017). Expression of the chemokine receptors CCR1 and CCR2B is up-regulated in peripheral blood B cells upon EBV infection and in established lymphoblastoid cell lines. Virology.

[28] Karpova M.B., Schoumans J., Blennow E., Ernberg I., Henter J.I., Smirnov A.F., Nordenskjöld M., Fadeel B. (2006). Combined spectral karyotyping, comparative genomic hybridization, and in vitro apoptyping of a panel of Burkitt’s lymphoma-derived B cell lines reveals an unexpected complexity of chromosomal aberrations and a recurrence of specific abnormalities in chemoresistant cell lines. Int. J. Oncol..

[29] Nagy N., Maeda A., Bandobashi K., Kis L.L., Nishikawa J., Trivedi P., Faggioni A., Klein G., Klein E. (2002). SH2D1A expression in Burkitt lymphoma cells is restricted to EBV positive group I lines and is downregulated in parallel with immunoblastic transformation. Int. J. Cancer.

[30] Pokrovskaja K., Ehlin-Henriksson B., Bartkova J., Bartek J., Scuderi R., Szekely L., Wiman K.G., Klein G. (1996). Phenotype-related differences in the expression of D-type cyclins in human B cell-derived lines. Cell Growth Differ..

[31] Tierney R.J., Steven N., Young L.S., Rickinson A.B. (1994). Epstein-Barr virus latency in blood mononuclear cells: Analysis of viral gene transcription during primary infection and in the carrier state. J. Virol..

[32] Bell A., Groves K., Kelly G.L., Croom-Carter D., Hui E., Chan A.T., Rickinson A.B. (2006). Analysis of Epstein-Barr virus latent gene expression in endemic Burkitt’s lymphoma and nasopharyngeal carcinoma tumour cells by using quantitative real-time PCR assays. J. Gen. Virol..

[33] Kholodnyuk I.D., Kozireva S., Kost-Alimova M., Kashuba V., Klein G., Imreh S. (2006). Down regulation of 3p genes, LTF, SLC38A3 and DRR1, upon growth of human chromosome 3-mouse fibrosarcoma hybrids in severe combined immunodeficiency mice. Int. J. Cancer.

[34] Expression Atlas Database—EMBL-EBI. https://www.ebi.ac.uk/gxa/home.

[35] Mellado M., Rodriguez-Frade J.M., Vila-Coro A.J., Fernandez S., Martin D.A., Jones D.R., Toran J.L., Martinez A. (2001). Chemokine receptor homo- or heterodimerization activates distinct signaling pathways. EMBO J..

[36] Sohy D., Yano H., de Nadai P., Urizar E., Guillabert A., Javitch J.A., Parmentier M., Springael J.Y. (2009). Hetero-oligomerization of CCR2, CCR5, and CXCR4 and the protein effects of “selective” antagonists. J. Biol. Chem..

[37] Proudfoot A.E., Uguccioni M. (2016). Modulation of Chemokine Responses: Synergy and Cooperativity. Front. Immunol..

[38] Huang Y., Chen H., Wang J., Bunjhoo H., Xiong W., Xu Y., Zhao J. (2013). Relationship between CCR2-V64I polymorphism and cancer risk: A meta-analysis. Gene.

[39] Liu G.X., Zhang X., Li S., Koiiche R.D., Sindsceii J.H., Song H. (2013). Monocyte chemotactic protein-1 and CC chemokine receptor 2 polymorphisms and prognosis of renal cell carcinoma. Tumour Biol..

[40] Narter K.F., Agachan B., Sozen S., Cincin Z.B., Isbir T. (2010). CCR2-64I is a risk factor for development of bladder cancer. Genet. Mol. Res..

[41] Ikuta K., Satoh Y., Hoshikawa Y., Sairenji T. (2000). Detection of Epstein-Barr virus in salivas and throat washings in healthy adults and children. Microbes Infect..

[42] Hadinoto V., Shapiro M., Sun C.C., Thorley-Lawson D.A. (2009). The dynamics of EBV shedding implicate a central role for epithelial cells in amplifying viral output. PLoS Pathog..

[43] Tugizov S.M., Berline J.W., Palefsky J.M. (2003). Epstein-Barr virus infection of polarized tongue and nasopharyngeal epithelial cells. Nat. Med..

[44] Shannon-Lowe C., Rowe M. (2011). Epstein-Barr virus infection of polarized epithelial cells via the basolateral surface by memory B cell-mediated transfer infection. PLoS Pathog..

[45] Tugizov S.M., Herrera R., Palefsky J.M. (2013). Epstein-Barr virus transcytosis through polarized oral epithelial cells. J. Virol..

[46] Buettner M., Meyer B., Schreck S., Niedobitek G. (2007). Expression of RANTES and MCP-1 in epithelial cells is regulated via LMP1 and CD40. Int. J. Cancer.

[47] Tugizov S., Herrera R., Veluppillai P., Greenspan J., Greenspan D., Palefsky J.M. (2007). Epstein-Barr virus (EBV)-infected monocytes facilitate dissemination of EBV within the oral mucosal epithelium. J. Virol..

[48] Shimakage M., Kimura M., Yanoma S., Ibe M., Yokota S., Tsujino G., Kozuka T., Dezawa T., Tamura S., Ohshima A. (1999). Expression of latent and replicative-infection genes of Epstein-Barr virus in macrophage. Arch. Virol..

[49] Savard M., Bélanger C., Tardif M., Gourde P., Flamand L., Gosselin J. (2000). Infection of primary human monocytes by Epstein-Barr virus. J. Virol..

